# An unusual presentation of immotile-cilia syndrome with azoospermia: Case report and literature review

**DOI:** 10.4103/0970-2113.56352

**Published:** 2009

**Authors:** Ramakant Dixit, Kalpana Dixit, Savita Jindal, K. V. Shah

**Affiliations:** *J. L. N. Medical College, Ajmer, India*; 1 *Department of Institute of Cardiology, B. J. Medical College, Ahmedabad, India*; 2 *Department of Chest Diseases and Tuberculosis, B. J. Medical College, Ahmedabad, India*

**Keywords:** Azoospermia, bronchiectasis, immotile-cilia syndrome, situs inversus

## Abstract

Immotile-cilia syndrome is a rare disorder characterized by chronic recurrent sino-pulmonary infection, impaired tracheobronchial clearance, situs inversus in about 50% of cases, and living but immotile spermatozoa of normal morphology in semen analysis. In this report, we describe an unusual presentation of immotile-cilia syndrome with azoospermia in a 32-year-old male patient. The diagnosis was based on history of recurrent respiratory tract infection, bronchiectasis, maxillary sinusitis, hypoplasia of frontal sinuses, dextrocardia with situs inversus, impaired nasal mucociliary clearance, etc. Semen analysis revealed azoospermia without any evidence of obstruction in epididymides or vas deference. Normal spermatogenesis was seen on testicular biopsy.

## INTRODUCTION

Immotile-cilia syndrome (ICS) is a rare disorder with a prevalence of about one in 30,000 among the general population.[1] It is probably transmitted by an autosomal recessive gene with incomplete penetrance. It is characterized by ultra-structural abnormalities in the cilia of respiratory tract and tail of spermatozoa. This leads to chronic recurrent sino-pulmonary infection, impaired tracheobronchial clearance, situs inversus in about 50% of cases, and living but immotile spermatozoa of normal morphology.[1–3] Kartagener syndrome is included as a subgroup or part of this syndrome.

The present communication describes a case of ICS with azoospermia, a variation from the usual presentation of ICS, in view of the rarity of this situation and lack of such reports in recent literature.

## CASE REPORT

A 32-year-old male farmer came to us for cough with purulent expectoration, low-grade fever, nasal discharge, and headache for the last two months. He had history of similar illness off and on since early childhood. He gave history of hospitalization three times for sino-pulmonary infection, as well as intermittent antibiotic administration. He took antituberculosis therapy for six months at the age of 20 years on account of slowly resolving pneumonitis. He is a nonsmoker and had never consumed alcohol. He also denied history suggestive of otitis media, gastrointestinal disease, cardiac failure, or genital infection.

There was no family history of recurrent sino-pulmonary infection. He was married for the last seven years but had no issue despite history of normal puberty, libido, and potency. Infertility workup of his wife did not reveal any abnormality.

On examination, the patient appeared pale with digital clubbing. The apex beat was in the right fifth intercostal space in midclavicular line with area of cardiac dullness on the right side. The liver was situated on the left side, with tympanic stomach resonance detected on the right side. Respiratory system examination revealed tenderness over paranasal sinuses and coarse crackles over left infrascapular, infra-axillary, and lower mammary regions. He appeared eugonadal with normal-size gonads and right testis placed at a lower level than the left.

**Figure 1:**
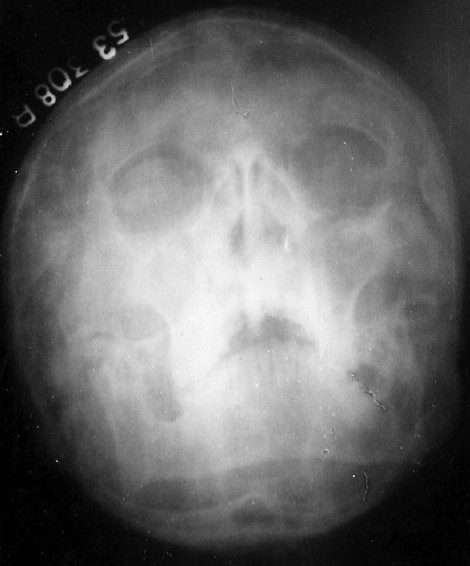
X-ray paranasal sinus showing maxillary sinusitis and bilateral hypoplastic frontal sinuses

**Figure 2:**
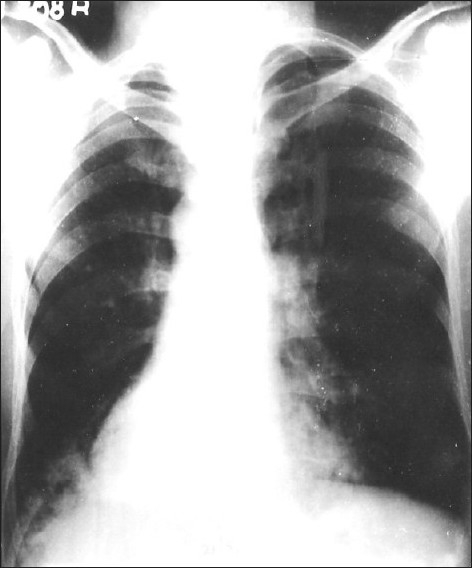
X-ray chest, PA view, showing dextrocardia and inhomogeneous infiltrate at both the lower zones in paracardiac region

**Figure 3:**
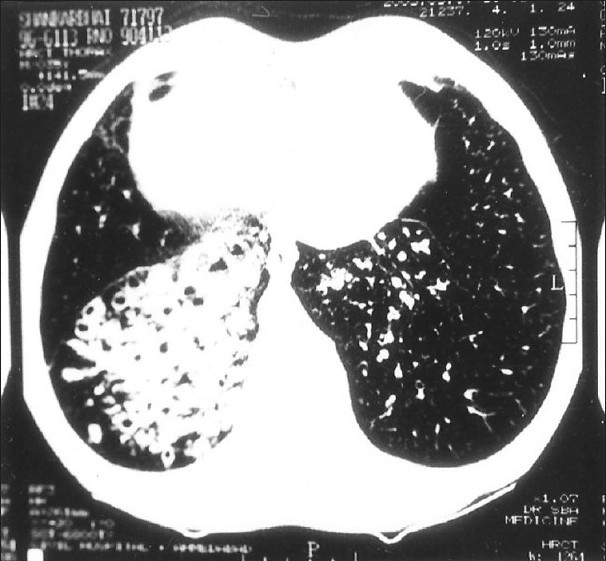
CT scan chest showing bronchiectatic changes and consolidation at lower lobes (more on right side)

His investigations revealed hemoglobin of 11.5 g% with normal blood counts, blood biochemistry, urine analysis, etc. Serological tests for HIV and HBsAg were nonreactive. Sputum smear microscopy was negative for acid-fast bacilli, fungi, and other microorganisms; and pyogenic culture was also sterile. Pulmonary function tests revealed mild obstructive type of ventilatory defect. Nasal mucociliary clearance measured by the saccharin test was more than 60 minutes (normal <35 minutes). Semen analysis revealed a volume of 3 mL with complete azoospermia on two occasions. X-ray paranasal sinus showed bilateral hypoplastic frontal sinuses and maxillary sinusitis, more on the right side [Figure 1]. X-ray chest, PA view, revealed right-sided heart and bilateral inhomogeneous infiltrates in lower zones [Figure 2]. On CT scan thorax, there was dextrocardia, consolidation involving posterior basal segments on the right side and lingular segment on the left side, associated with bronchiectatic changes [Figure 3]. Barium meal and follow-through examination further confirmed situs inversus totalis.

Bronchoscopy and electron microscopy of nasal mucosal biopsy could not be performed. Testicular biopsy showed normal spermatogenesis and normal Leydig cells without any tubular blockage. On vasography, there was no obstruction in vas deference and ejaculatory ducts. There was no obstruction in the epididymis, and aspirates from epididymis also showed lack of sperms on light microscopy examination. The sino-pulmonary infection was managed conservatively, and the patient was referred to an infertility specialist.

## DISCUSSION

The triad of situs inversus, bronchiectasis, and sinusitis has carried Kartagener's name since he reported four cases in 1933.[4] Although Siewart[5] first described this condition in 1904, the credit of recognizing the etiological correlation between the elements of the triad goes to Kartagener, whose name has been associated with this condition. Up to 1955, only 104 cases of this syndrome were reported in the literature; subsequently, more cases of this condition were recognized, including a few from our country.[6,7]

The issue of fertility was not addressed in the initial published reports of patients with Kartagener syndrome, until Arge[8] reported three male patients with this syndrome having immotile spermatozoa and sterility. In 1976, Afzelius noted a lack of dynein arm in the sperm and cilia of four subjects; out of them, three had Kartagener syndrome.[9] In 1977, Eliasson and associates[2] proposed that what they called the immotile-cilia syndrome (ICS) was responsible for chronic airway infection and male sterility in a wide variety of persons. Following these original reports, rather landmark observations, a new syndrome, called ICS, was recognized and applied to many patients with or without Kartagener syndrome.[10]

The cause of this condition was found to be a congenital defect in the ultra-structure of the cilia in the form of lack of dynein arms. In addition, the definition was broadened to include patients with other ciliary abnormalities, including transposition of ciliary microtubules, defective radial spokes, and absence of inner but preservation of outer dynein arms.[11] Other electron microscopic abnormalities that are reported include missing nexin link, compound cilia, supernumerary, absent or incomplete microtubules, lack of ciliary orientation, and abnormal attachment of the dynein arms, etc.[12,13]

In the recent years, the term ICS has been replaced by the term primary ciliary dyskinesia. This replacement reflects the findings that the cilia of many patients do move, albeit in an uncoordinated fasion.[14] Whatever may be the defect, whether immotility or inappropriate motility (insufficient beating pattern), because cilia occurs mainly in the respiratory tract and genital tract, the clinical symptoms of ICS are mostly chronic sinusitis, bronchitis, bronchiectasis, and male sterility. The situs inversus, which occurs in approximately 50% of patients with this disorder, is possibly caused by an inability of embryonic cilia to shift the heart to the left side; however, the exact genetic link between ciliary dyskinesia and situs inversus is still not clear.[11] It is also suggested that the absence of dynein in the axoneme is probably part of a diffuse genetic defect, which may extend to cytoplasmic, nonaxonemal dynein and lead to a disturbance of various microtubule-dependent cell activities. This also seems true as there are reports of ICS associated with hepatic parenchymal steatosis,[15] retinitis pigmentosa due to abnormal cilia of retinal pigment epithelium,[16] polysplenia, extra-hepatic biliary atresia,[17] etc., suggesting a single dysmorphogenetic process.

The suggested criteria used for diagnosis of ICS include clinical signs of chronic bronchitis and rhinitis from early infancy, combined with one or more of the following additional symptoms:[18] 1) situs inversus in the patient or in sibling, with similar clinical signs; 2) living but immotile spermatozoa of normal appearance on semen analysis; 3) transbronchial clearance absent or markedly depressed; 4) cilia with characteristic ultra-structural defect. Our case fulfills the diagnostic criteria for ICS with variation in view of ‘complete azoospermia’ despite normal spermatogenesis and without any obstruction in the ducts. This situation is extremely rare and has not been reported in recent literature. Bashi *et al.,*[19] reported a similar case in 1988 from Riyadh, Saudi Arabia. Gill *et al.,*[20] reported a case of Kartagener syndrome along with left empyema thoracis and azoospermia in 1996 from Varanasi, India. In the latter, the authors attributed azoospermia in their case to asthenozoospermia, oligozoospermia, or oligoasthenozoospermia. The possible explanation for azoospermia in our case could be failure of sperm transport due to combination of defective ciliary function in the epididymis and vas deferens and abnormal motility of the sperm itself. The other contributing factors could be lack of contraction of myoids in the walls of tubules and muscular elements in tunica albugenia.

The diagnosis of ICS in our case was based on clinico-radiological features and supported by impaired nasal mucociliary transport. The clinical presentation with chronic cough, bronchiectasis, maxillary sinusitis, hypoplasia of frontal sinus, and situs inversus along with infertility was sufficient enough to suspect ICS. The electron microscopic study to detect ultra-structural defects in ciliary apparatus could not be done due to lack of this facility at our center. Although electron microscopies of sperm tail and respiratory cilia are usually required for diagnostic confirmation in such cases, there may be no abnormality in some variants of primary ciliary dyskinasia.[21] Further, it is also observed that none of the ciliary defects observed in ICS are specific. They may all occur as secondary lesions or sporadically as varieties in otherwise healthy subjects.[12] We therefore conclude with the remark that ICS should be suspected on clinical grounds in individuals presenting with chronic cough, rhinorrhea, sinusitis, otitis media, obstructive lung disease, etc., along with male sterility and with or without situs inversus.
